# The effects of selected sedatives on basal and stimulated serum cortisol concentrations in healthy dogs

**DOI:** 10.7717/peerj.16955

**Published:** 2024-02-20

**Authors:** Adam Hunt, Shelly Olin, Jacqueline C. Whittemore, Alejandro Esteller-Vico, Cary Springer, Luca Giori

**Affiliations:** 1 Department of Small Animal Clinical Sciences, University of Tennessee, College of Veterinary Medicine, Knoxville, TN, United States of America; 2 Animal Emergency and Specialty Center, Knoxville, TN, United States of America; 3 University of Tennessee, College of Veterinary Medicine, Department of Biomedical and Diagnostic Sciences, TN, United States of America; 4 Research Computing Support, Office of Information Technology, University of Tennessee–Knoxville, Knoxville, TN, United States of America

**Keywords:** Hyperadrenocorticism, Hypoadrenocorticism, Cushings, Addisons, Cortisol, Sedation, Canine

## Abstract

**Background:**

Hormone assessment is typically recommended for awake, unsedated dogs. However, one of the most commonly asked questions from veterinary practitioners to the endocrinology laboratory is how sedation impacts cortisol concentrations and the adrenocorticotropic hormone (ACTH) stimulation test. Butorphanol, dexmedetomidine, and trazodone are common sedatives for dogs, but their impact on the hypothalamic-pituitary-adrenal axis (HPA) is unknown. The objective of this study was to evaluate the effects of butorphanol, dexmedetomidine, and trazodone on serum cortisol concentrations.

**Methods:**

Twelve healthy beagles were included in a prospective, randomized, four-period crossover design study with a 7-day washout. ACTH stimulation test results were determined after saline (0.5 mL IV), butorphanol (0.3 mg/kg IV), dexmedetomidine (4 µg/kg IV), and trazodone (3–5 mg/kg PO) administration.

**Results:**

Compared to saline, butorphanol increased basal (median 11.75 µg/dL (range 2.50–23.00) (324.13 nmol/L; range 68.97–634.48) *vs* 1.27 µg/dL (0.74–2.10) (35.03 nmol/L; 20.41–57.93); *P* < 0.0001) and post-ACTH cortisol concentrations (17.05 µg/dL (12.40–26.00) (470.34 nmol/L; 342.07–717.24) *vs* 13.75 µg/dL (10.00–18.90) (379.31 nmol/L; 275.96–521.38); *P* ≤ 0.0001). Dexmedetomidine and trazodone did not significantly affect basal (1.55 µg/dL (range 0.75–1.55) (42.76 nmol/L; 20.69–42.76); *P* = 0.33 and 0.79 µg/dL (range 0.69–1.89) (21.79 nmol/L; 19.03–52.14); *P* = 0.13, respectively, *vs* saline 1.27 (0.74–2.10) (35.03 nmol/L; 20.41–57.93)) or post-ACTH cortisol concentrations (14.35 µg/dL (range 10.70–18.00) (395.86 nmol/L; 295.17–496.55); (P = 0.98 and 12.90 µg/dL (range 8.94–17.40) (355.86 nmol/L; 246.62–480); *P* = 0.65), respectively, *vs* saline 13.75 µg/dL (10.00–18.60) (379.31 nmol/L; 275.86–513.10).

**Conclusion:**

Butorphanol administration should be avoided prior to ACTH stimulation testing in dogs. Further evaluation of dexmedetomidine and trazodone’s effects on adrenocortical hormone testing in dogs suspected of HPA derangements is warranted to confirm they do not impact clinical diagnosis.

## Introduction

Definitive diagnosis of hypothalamic-pituitary axis (HPA) disorders depends on accurate adrenal function testing in conjunction with compatible clinical signs, biochemical changes, and ultrasonographic findings. The adrenocorticotropic hormone (ACTH) stimulation test is commonly used in diagnosis of both hyperadrenocorticism (HAC) and hypoadrenocorticism (Behrend et al., 2013; Carotenuto et al., 2019; Guzmán Ramos et al., 2022). Basal cortisol concentration often is used as a screening test for hypoadrenocorticism and increasingly is used for monitoring treatment of HAC (Guzmán Ramos et al., 2022; Macfarlane, Parkin & Ramsey, 2016). Hormone assessment is typically recommended for awake, unsedated dogs. However, one of the most commonly asked questions from veterinary practitioners to the Diagnostic Endocrinology Laboratory at our institution is how sedation impacts cortisol concentrations and the ACTH stimulation test (personal experience, LG, AE).

Dogs can receive sedation as part of fear-free handling and to facilitate accurate and safe diagnostic imaging. Abdominal ultrasound findings such as adrenal gland changes, hyperechoic hepatomegaly, cholecystitis, and gallbladder mucocele could prompt adrenal gland function testing (Kim et al., 2017). The effects of sedation on post-ACTH stimulation test (post-ACTH) hormone concentrations are unknown. As a result, adrenal testing can be delayed–potentially increasing costs, heightening patient anxiety, and decreasing owner compliance. Alternatively, dogs could be tested despite receiving anxiolytic or sedative drugs, which could lead to misdiagnosis if the drugs meaningfully impact post-ACTH results (Behrend et al., 2013).

Butorphanol (Torbugesic®, Zoetis Inc. Kalamazoo, MI), dexmedetomidine (Dexdomitor®, Zoetis Inc., Kalamazoo, MI, USA), and trazodone (Teva Pharmaceuticals, Parsippany, NJ, USA) commonly are used in dogs for sedative and/or anxiolytic effects. However, the effects of these drugs on the HPA are not completely understood. For example, an 80 µg/kg dose of dexmedetomidine in dogs reduced basal and post-ACTH cortisol concentrations enough to impact clinical decision making (Maze et al., 1991). Although a 10 µg/kg dose did not significantly impact basal cortisol concentrations (Restitutti et al., 2012), the effects on post-ACTH cortisol concentrations are unknown. Butorphanol increases basal cortisol concentrations in dogs (Fox et al., 1998; Ambrisko, Hikasa & Sato, 2005), whereas trazodone decreases basal cortisol concentrations in dogs and humans (Morris et al., 2020; Manthey et al., 2011). The effect of either drug on post-ACTH cortisol concentrations in dogs is unknown (Fox et al., 1998; Ambrisko, Hikasa & Sato, 2005; Morris et al., 2020; Manthey et al., 2011).

The objective of this study was to determine the effects of butorphanol, dexmedetomidine, and trazodone on basal and post-ACTH cortisol concentrations. We hypothesized that trazodone and dexmedetomidine would decrease and butorphanol would increase basal and post-ACTH cortisol concentrations, respectively.

## Materials and Methods

The study was a prospective, controlled, randomized, repeated measure four-treatment, four-period crossover design. The Institutional Animal Care and Use Committee at the University of Tennessee, College of Veterinary Medicine approved this work (protocol number 2758-0420).

### Animals

Twelve Beagle dogs from the University of Tennessee, College of Veterinary Medicine colony were enrolled over a 4-week period. Inclusion and exclusion criteria were established *a priori*. To be eligible for inclusion, dogs had to be clinically healthy based on a review of medical records from the previous 30 days, lack of clinically relevant abnormalities on CBC and biochemical profile performed annually as part of colony wellness, no history of endocrine disease, and normal physical examination at the time of study enrollment. Dogs that received medications known to affect the HPA within 30 days of enrollment were excluded. All physical examinations were completed by one of two investigators (AH, SO). The study was completed in the animals’ housing areas. Following sedation, pulse and respiratory rate were monitored every 5 min until a dog could maintain sternal recumbency, and then every 30 min. Body temperature was measured every 30 min.

Throughout the study, the dogs were fed their normal maintenance diet twice daily and housed in individual runs. Dogs had an enrichment schedule including daily music, twice weekly scents and toy rotation, ice +/− broth five times per week, frozen wet food toy weekly, and a minimum of group play three times per week. General anesthesia or euthanasia were not part of the study design; no dogs met the criteria for euthanasia prior to the planned end of the study (*i.e*., severe illness, cardiopulmonary arrest). At the study conclusion, all dogs were returned to the colony until their eventual adoption.

### Treatment groups

Prior to randomization, a rotating treatment schedule was made (Table S1). A random number generator was used to assign dogs to groups of three. Each group rotated through four treatments as per the randomized treatment schedule, with a 7-day washout between treatments: butorphanol (0.3 mg/kg IV), dexmedetomidine (4 µg/kg IV), trazodone (3–5 mg/kg PO, rounded to the nearest 50 mg tablet size), or saline (0.5 mL IV). The 7-day washout time frame was selected to allow standardization of timing of sampling and avoid potential for intra-weekend variation in colony activities and enrichment to affect results. The degree of sedation was evaluated by one of two investigators (AH, SO) and described subjectively as none, mild, moderate, or marked. Any dog with nausea persisting through the end of sample collection was administered maropitant (1 mg/kg, IV, Cerenia®; Zoetis Inc., Kalamazoo, MI, USA). Drug dosages were calculated based on the dog’s weight on the morning of the selected intervention.

### ACTH stimulation and sample collection

Dogs were fasted a minimum of 12 h. Sample collection (phlebotomy) was performed in the morning. Basal blood sampling was performed 90 min after butorphanol (Fox et al., 1998), trazodone (Ashraf, Shariati & Zamanpor, 2014), and saline administration, with sampling performed 120 min after dexmedetomidine administration. Sampling intervals were based on projected time of maximal effect on basal cortisol based on limited prior data (Maze et al., 1991; Restitutti et al., 2012; Fox et al., 1998; Ambrisko, Hikasa & Sato, 2005; Morris et al., 2020; Manthey et al., 2011; Ashraf, Shariati & Zamanpor, 2014). When possible, phlebotomy to collect blood for hormone analysis was performed using the jugular vein. Following phlebotomy, dogs were administered cosyntropin (5 μg/kg, IV, Cortrosyn^TM^; Amphastar Pharma, Rancho Cucamonga, CA, USA) *via* cephalic vein, followed by collection of a post-ACTH blood sample 1 h later (Carotenuto et al., 2019; Guzmán Ramos et al., 2022). Blood was allowed to clot at room temperature and then centrifuged at 2,200 × *g* for 15 min to separate serum. Serum was stored at −20 °C pending sample analysis. After blood samples were collected, atipamezole (Antisedan; Zoetis Inc., Kalamazoo, MI, USA) was given at the clinician’s discretion to dogs that received dexmedetomidine.

### Hormone analysis

Hormone analyses were performed en masse on thawed serum samples. Cortisol was measured once using a chemiluminescence immunoassay system (Immulite 2000 XPi; Siemens Healthcare Diagnostics Products Ltd., Los Angeles, CA, USA) with a specific cortisol reagent kit (K2KCO6; Siemens Healthcare Diagnostics Products Ltd., Los Angeles, CA, USA). The analyzer performs 12 replicate measurements for each sample, discards the highest and lowest sample, and reports the mean of the 10 remaining measurements. Hormone measurements were performed in accordance with the manufacturer’s instructions by laboratory personnel blinded to dog identity, treatment intervention, and time point.

### Statistical analysis

*A priori* sample size calculation found a minimum of 12 subjects required to detect a difference of 5 µg/dL in post-ACTH stimulation cortisol concentration with an alpha of 0.05 and a beta of 0.8.

A prospective, controlled, randomized, repeated measure four-treatment, four-period crossover design was used to evaluate changes in basal and post-ACTH cortisol concentration. Each response was analyzed using a repeated-measures mixed-model analysis of variance (ANOVA) with treatment set as a fixed factor and dog within sequence as a random factor. Sex was included as a covariate. Basal cortisol was rank-transformed to meet statistical assumptions regarding normality. Sequence and period effects were tested. All models used Kronecker product variance/covariance structures. *Post-hoc* comparisons used a Tukey-Kramer correction. Normality of residuals were evaluated using a Shapiro-Wilk test for normality and quantile-quantile plots (QQ plots). Equality of treatment variances was tested with Levene’s equality of variances test. All statistical assumptions regarding normality and equality of variances were met.

Dogs with cortisol concentrations outside laboratory reference intervals (RIs) were numerated; the reference intervals are specific to the sex and reproductive status of each dog (see raw data file). The number of dogs with post-ACTH cortisol concentrations ≥20 µg/dL (Korchia & Freeman, 2021) also was numerated. Statistical analyses were performed using commercial software (SAS software, version 9.4, Release TS1M6; SAS Institute, Cary, NC, USA). Statistical significance was defined as a *P* value < 0.05.

## Results

### Animals

Of the 12 dogs enrolled in the study, there were seven neutered males and five spayed females. All dogs were 5 years old with a mean (±SD) body weight of 11.6 ± 2.0 kg and median body condition score of 7/9 (range 5–8). All 12 dogs receiving butorphanol had mild sedation. Of these, ptyalism was noted in five dogs and three dogs were administered maropitant after all sampling was completed. The degree of sedation after dexmedetomidine administration was variable (none, two dogs; mild, three dogs; moderate, five dogs; marked, two dogs). Three dogs had a single vomiting event following dexmedetomidine administration, but none appeared nauseated or received maropitant. Atipamezole was not given to any dog. For trazodone, five dogs appeared unaffected by sedation and seven dogs were mildly sedated. No nausea or vomiting was noted for any dog after receiving trazodone. All 12 dogs were normal and there were no adverse events after receiving saline.

### Cortisol assays

All results were included in analysis and results of cortisol assays are presented in Table 1. There were no significant effects of treatment by sequence (*P* = 0.57 and *P* = 0.63, respectively) or week number (*P* = 0.37 and *P* = 0.20, respectively) on basal or post-ACTH cortisol concentrations. Significant effects of treatment were noted, with most significant effects reflecting the influence of butorphanol.

**Table 1:** Median (range) basal and post-ACTH stimulation serum cortisol concentrations for healthy dogs (*n* = 12) after receiving saline, butorphanol, dexmedetomidine, and trazodone.

Saline		Butorphanol	*P*-value	Dexmedetomidine	*P*-value	Trazodone	*P*-value
Cortisol (µg/dL)							
Basal	1.27 (0.74–2.10)^bc^	11.75 (2.50–23.00)^a^	<0.0001	1.55 (0.75–1.55)^b^	0.33	0.79 (0.69–1.89)^c^	0.79
Post-ACTH	13.75 (10.00–18.60)^b^	17.05 (12.40–26.00)^a^	<0.0001	14.35 (10.70–18.00)^b^	0.98	12.90 (8.94–17.40)^b^	0.65
Cortisol (nmol/L)							
Basal	35.03 (20.69–57.93)^bc^	324.14 (68.97–634.48)^a^	<0.0001	42.76 (20.69–42.76)^b^	0.33	21.79 (19.03–52.14)^c^	0.79
Post-ACTH	379.31 (275.86–513.10)^b^	470.34 (342.07–717.24)^a^	<0.0001	395.86 (295.17–496.55)^b^	0.98	355.86 (246.62–1438.34)^b^	0.65

**Note:**

Significance was defined as *P* < 0.05. Values within a row that do not share a superscript letter differed significantly based on *post-hoc* analysis.

After saline administration, no dog had increased basal cortisol concentration. Post-ACTH stimulation, three dogs in the saline group had cortisol concentrations above the RI (1.1–1.2 times > RI) but <20 µg/dL (<551.72 nmol/L). After butorphanol administration, 10 dogs had increased basal cortisol concentrations (1.4–4 times > RI). Post-ACTH stimulation, seven dogs receiving butorphanol with cortisol concentrations above the RI (1.1–1.7 times > RI) and two dogs had cortisol concentrations >20 µg/dL (>551.72 nmol/L). Regarding dexmedetomidine, no dog had an increased basal cortisol concentration. Post-ACTH stimulation, four dogs receiving dexmedetomidine had cortisol concentrations above the RI (1.03–1.08 times > RI) but <20 µg/dL (<551.72 nmol/L). Post-trazodone, no dog had increased basal cortisol concentration, but two dogs had post-ACTH cortisol concentrations above the RI (1.1–1.2 times > RI) but <20 µg/dL (<551.72 nmol/L).

## Discussion

Butorphanol administration causes clinically and statistically significant increases in basal and post-ACTH cortisol concentrations in healthy dogs. Following butorphanol administration, basal cortisol concentrations were above the currently used cut-off for therapeutic monitoring of trilostane (>5.5 µg/dL; >151.75 nmol/L) (Macfarlane, Parkin & Ramsey, 2016) for 83% of subjects. Post-ACTH cortisol values in 16% of subjects exceeded a widely used diagnostic cut-off for hyperadrenocorticism (>20 µg/dL; >551.72 nmol/L) (Korchia & Freeman, 2021). In contrast, neither dexmedetomidine nor trazodone administration at the dosages and time points evaluated in this study altered basal or post-ACTH cortisol concentrations.

Increases in basal cortisol concentrations after butorphanol administration are consistent with results of previous studies (Fox et al., 1998; Ambrisko, Hikasa & Sato, 2005). Additionally, butorphanol administration significantly increased post-ACTH cortisol concentrations. The authors hypothesize that possible mechanisms for this change include increased hormone release, transcellular movement, or decreased hormone clearance. Increased cortisol concentrations following butorphanol administration have been hypothesized to be partly due to noxious psychogenic effects (dysphoria) (Fox et al., 1998, 1994). In humans, dysphoria after butorphanol administration can cause an anxiety-like state arising from or accompanied by headache, dizziness, and confusion (Fox et al., 1998). One study supported this theory by documenting that the increase in basal cortisol concentration following butorphanol administration in dogs was suppressed with the induction of general anesthesia and loss of consciousness, but cortisol concentrations increased again when consciousness returned while butorphanol was still in circulation (Fox et al., 1998). Alternatively, administration of butorphanol might mimic the effect of endogenous opioids, causing release of catecholamines and increasing cortisol concentrations (Ambrisko, Hikasa & Sato, 2005). Dogs had the most nausea in our study following butorphanol administration with 42% of dogs experiencing ptyalism. Nausea-associated-stress could explain the higher cortisol concentrations in this group of dogs. However, 25% of dogs had a single episode of vomiting after receiving dexmedetomidine but all had baseline cortisol concentrations within the RI. This suggests that nausea or vomiting alone is not the only explanation for cortisol increases after butorphanol administration.

Benefits of the ACTH stimulation test as compared to the low dose dexamethasone test, include rapid testing time and decreased effect of stress on results (Behrend et al., 2013). Although sensitivity and specificity of ACTH stimulation testing for hyperadrenocorticism vary among populations and disease states, results often are used to dictate potential therapeutic interventions (Behrend et al., 2013). All dogs treated with saline, dexmedetomidine, or trazodone in this study had post-ACTH cortisol concentrations <20 μg/dL (<551.72 nmol/L). In contrast, 7/12 (58%) healthy dogs administered with butorphanol had cortisol concentrations between 16–20 μg/dL (441.38–551.72 nmol/L) and 2/12 (16.6%) had post-ACTH cortisol concentrations >20 μg/dL (>551.72 nmol/L). These differences could have resulted in misdiagnosis of hyperadrenocorticism when compared to the laboratory-provided RI or the commonly used clinical decision-making threshold of >20 μg/dL (>551.72 nmol/L) (Korchia & Freeman, 2021). Baseline cortisol concentrations prior to trilostane or 3 h post-trilostane administration increasingly are used for therapeutic monitoring of dogs with hyperadrenocorticism (Macfarlane, Parkin & Ramsey, 2016). Given significant increases in basal cortisol in healthy dogs after butorphanol administration in this study, use of butorphanol prior to collection of pre-trilostane or 3 h-post trilostane unstimulated cortisol concentration measurements could result in inappropriate dosage escalation. As a result, butorphanol administration should be avoided during cortisol determination.

Although the ACTH stimulation test is the definitive test for hypoadrenocorticism, baseline cortisol concentrations are widely used to rule out the disease (Guzmán Ramos et al., 2022). However, an ACTH stimulation test is recommended for dogs with baseline cortisol concentrations ≤2 μg/dL. Of dogs receiving saline, dexmedetomidine, and trazodone in this study, 11/12 (92%), 10/12 (83%) and 12/12 (100%), respectively, had baseline cortisol concentrations ≤2 μg/dL (≤55.17 nmol/L). In contrast, butorphanol administration resulted in 0/12 dogs with a basal cortisol concentration ≤2 μg/dL (<55.17 nmol/L). Further investigation is needed to understand if dogs with hypoadrenocorticism have increased cortisol concentration following butorphanol administration. Theoretically, dogs with hypoadrenocorticism lack adrenal reserve and do not have the capacity to increase cortisol concentration (Guzmán Ramos et al., 2022). However, butorphanol administration could still impact cortisol concentration if it alters cortisol clearance.

Our study population had a significantly lower median basal cortisol concentration than previously found for dogs without hypoadrenocorticism (Hauck et al., 2020; Bovens et al., 2014). As noted above, most dogs in this study had basal cortisol concentrations ≤2 μg/dL (<55.17 nmol/L) after saline administration (median 1.27, range 0.74–2.10 μg/dL; 35.03, 20.41–57.93 nmol/L). There is evidence that cortisol concentrations increase when a dog is placed in a novel environment (Fox et al., 1998). Because study participants were purpose bred colony dogs whose evaluations were performed within or adjacent to their normal housing environment and are used regularly for both teaching and research purposes, their stress levels could have been lower than those of clinical patients.

There is evidence that various doses of dexmedetomidine have different effects on the HPA (Maze et al., 1991; Restitutti et al., 2012). Anesthetic doses of dexmedetomidine in dogs (80 µg/kg) reduced basal and post-ACTH cortisol concentrations (Maze et al., 1991), whereas a dose of 10 µg/kg dexmedetomidine did not significantly impact basal cortisol concentration (Restitutti et al., 2012). Dexmedetomidine at a dose of 4 µg/kg did not significantly decrease basal cortisol concentrations in the present study as hypothesized. Future studies could evaluate the effects of various dosing regimens and alternative administration routes (*i.e.,* transmucosally).

Trazodone was the only oral sedative used in this study and *per os* is the most common administration route in clinical practice. Although trazodone has been reported to decrease basal cortisol concentrations in dogs when administered prior to a stressful event (Morris et al., 2020), it did not significantly affect basal or post-ACTH cortisol concentrations in the present study. Dogs in the present study had lower median basal cortisol concentrations compared to prior publications (Hauck et al., 2020; Bovens et al., 2014), which could have limited the study’s statistical power to detect a decrease in cortisol post-trazodone administration. Conversely, differences in dosing regimen could have contributed to discordant results among studies. Dogs in the present study received a single, lower dose (3–5 mg/kg) of trazodone on the morning of the study. Following administration, five dogs did not have clinically appreciable sedation and seven dogs were only mildly sedated. Dogs in the previous study (Morris et al., 2020) were administered two doses of trazodone (the night prior and morning of), instead of one, and received a higher trazodone dose (5–10 mg/kg). In a third study, dogs receiving a single morning dose of trazodone (8–10 mg/kg PO) had significantly decreased post-ACTH cortisol concentrations when the ACTH stimulation test was started 1-h post-sedation (Brown et al., 2023). It is possible that discordant hormonal findings among studies are due to differences in clinical sedation or dosage. It is also possible that the maximal hormonal effect of trazodone is not at 90 min, when hormone concentrations were measured in the present study. There can be individual variability in the time to reach maximum plasma concentration (T_max_) (Jay et al., 2013). More work is needed to clarify the effect of trazodone dosing and repeated administration on cortisol concentrations, as well as correlations with clinical effects and plasma concentrations.

The biggest limitation of this study is the use of a small number (*n* = 12) of healthy animals of the same breed and age with no signs of HPA dysfunction. Dogs typically undergo ACTH stimulation due to clinical and biochemical signs of HPA dysregulation. They also are of varied breed, ages, and with varying comorbidities–none of which were assessed in the present study. Future studies evaluating the effects of sedation on dogs of differing ages, breeds, and with HPA dysfunction are needed. All dogs received a single dose of each drug with a single time point for sampling after drug administration. The amount of time between sedation and basal hormone sampling was extrapolated from previous studies documenting when serum cortisol was most affected by the drug. However, this timing might not correlate with timing in clinical patients. Results might differ if hormones are measured at different time points after drug administration. It is also likely that different dosages, combination protocols, or repeated drug dosages would produce different results. Finally, the laboratory provided RIs used for this study were based on a diverse dog population and breed-specific RIs are not available.

The selection of the 90 and 120-min intervals for basal sample collection was based on an estimated time that a change in basal cortisol concentration might be seen following each sedative administration and was extrapolated from the known literature. However, there is very limited information available about the effect(s), if any, and duration of effect(s) from sedative administration on cortisol concentration, and it is difficult to compare studies due to variations in dosage and route of administration. Additionally, in general, it is unknown if the biological effects of sedative administration on cortisol concentration correlate with clinical sedation, plasma drug concentration, or other changes in the dog. It is possible that the maximal impact of sedation administration on adrenal function, if any, is delayed or unrelated to clinical sedative effects or plasma drug concentration. Measurement of plasma drug concentrations could have helped clarify the mechanism for significant effects, but they were not collected due to financial limitations. Further work is needed to clarify if cortisol concentration is impacted at time points other than 90 and 120 min post-administration, as well as by various dosages and routes of sedative administration.

Three dogs exhibited a marginal increase in post-ACTH cortisol levels above their respective RIs after receiving saline solution. One plausible explanation is that these cortisol concentrations could be considered normal for these specific individuals and fall within the scope of biological variability unique to each dog. However, it is important to note that the study did not include the execution of five ACTH stimulation tests to establish the homeostatic cortisol set point for each individual (Gal et al., 2017).

Furthermore, it’s possible that the observed marginal increases in cortisol concentrations might be attributed to analytical variability rather than biological fluctuations. In a recent study, the chemiluminescence immunoassay system employed in this research underwent assessment of cortisol measurement variability (Korchia & Freeman, 2021). Depending on the chosen interpretation threshold (*e.g*., 1.4 *vs* 20 μg/dL; 38.62 *vs* 551.72 nmol/L), the potential analytical variability ranged from 30% to 20%, respectively. In practical terms, this means that the true hormone concentration in the dog could fluctuate within a ± 30% range (*i.e*., 0.98 to 1.82 μg/dL; *i.e*., 27.03 to 50.20 nmol/L) when using a threshold of 1.4 μg/dL (38.62 nmol/L). In our study, the elevations above the RI (15.1 μg/dL; 416.55 nmol/L) observed in these three dogs were all within the range of analytical variability (19.6 μg/dL; 540.69 nmol/L), therefore considered not significant.

These observed post-saline cortisol values could have been influenced by the physiological stress response triggered by handling and restraint. However, this seems less likely as these dogs are purpose-bred colony dogs. Although no clinical or biochemical indications supported hyperadrenocorticism at the study’s time, the potential for early-stage disease couldn’t be entirely ruled out. Abdominal imaging was not conducted, and the dogs weren’t reevaluated after the study’s conclusion. Nonetheless, to the best of the authors’ knowledge, no animal that participated in the study subsequently displayed clinical signs consistent with hyperadrenocorticism.

## Conclusions

In conclusion, administration of a single dose of butorphanol (0.3 mg/kg IV) significantly increased basal and post-ACTH serum cortisol concentrations in healthy dogs. There was no significant effect from administration of a single dose of dexmedetomidine (4 µg/kg IV) or trazodone (3–5 mg/kg PO). However, caution should still be used when interpreting ACTH stimulation results after dexmedetomidine or trazodone is administered because this was a study of healthy Beagle dogs, with a single selected dose of these drugs given. Prospective studies are needed to evaluate the impact of commonly used sedation protocols and repeated dosing of sedatives and anxiolytics in dogs with adrenal disease prior to their use in clinical patients undergoing interrogation of the HPA.

## Supplemental Information

10.7717/peerj.16955/supp-1 Supplemental Information 1 Author checklist.

10.7717/peerj.16955/supp-2 Supplemental Information 2 Drug Allocation Schedule.

10.7717/peerj.16955/supp-3 Supplemental Information 3 Median (range) basal and post-ACTH stimulation serum cortisol concentrations for healthy dogs (*n* = 12) after receiving saline, butorphanol, dexmedetomidine, and trazodone in a crossover design.

## Additional Information and Declarations

### Data Availability

The following information was supplied regarding data availability: The raw data is available in the Supplemental File.
