# Supplementary material for: The effects of selected sedatives on basal and stimulated serum cortisol concentrations in healthy dogs
Source: PeerJ. 2024 Feb 20;12:e16955. doi: 10.7717/peerj.16955 (PMC10885794; doi:10.7717/peerj.16955)
Supplement: Supplemental Information 2 [file peerj-12-16955-s002.docx]

**Supplemental Table 1: Drug Allocation Schedule**

| **Dog Number** | **Week** | **Day 1** | **Day 2** | **Day 3** | **Day 4** |
| --- | --- | --- | --- | --- | --- |
| 1, 2, 3 | 1 | Saline |  |  |  |
| 4, 5, 6 |  |  | Dexmedetomidine |  |  |
| 7, 8, 9 |  |  |  | Butorphanol |  |
| 10, 11, 12 |  |  |  |  | Trazodone |
|  |  |  |  |  |  |
| 1, 2, 3 | 2 | Dexmedetomidine |  |  |  |
| 4, 5, 6 |  |  | Butorphanol |  |  |
| 7, 8, 9 |  |  |  | Trazodone |  |
| 10, 11, 12 |  |  |  |  | Saline |
|  |  |  |  |  |  |
| 1, 2, 3 | 3 | Butorphanol |  |  |  |
| 4, 5, 6 |  |  | Trazodone |  |  |
| 7, 8, 9 |  |  |  | Saline |  |
| 10, 11, 12 |  |  |  |  | Dexmedetomidine |
|  |  |  |  |  |  |
| 1, 2, 3 | 4 | Trazodone |  |  |  |
| 4, 5, 6 |  |  | Saline |  |  |
| 7, 8, 9 |  |  |  | Dexmedetomidine |  |
| 10, 11, 12 |  |  |  |  | Butorphanol |
